# A Cooperative Learning Intervention to Promote Social Inclusion in Heterogeneous Classrooms

**DOI:** 10.3389/fpsyg.2020.586489

**Published:** 2020-12-22

**Authors:** Nina Klang, Ingrid Olsson, Jenny Wilder, Gunilla Lindqvist, Niclas Fohlin, Claes Nilholm

**Affiliations:** ^1^Department of Education, Uppsala University, Uppsala, Sweden; ^2^Department of Special Education, Faculty of Social Sciences, Stockholm University, Stockholm, Sweden; ^3^Association of Cooperative Learning for Teachers, Stockholm, Sweden

**Keywords:** inclusion, cooperative learning, teachers, children with special needs, intervention

## Abstract

Concerning challenges with the social inclusion of children with special educational needs (SEN), it is imperative to evaluate teacher interventions that promote social inclusion. This study aimed to investigate the effects of cooperative learning (CL) intervention on social inclusion. In addition, it was investigated to what degree CL implementation affected the outcomes. Fifty-six teachers of 958 fifth-grade children were randomly selected to intervention and control groups upon recruitment to the study. The intervention teachers received training and coaching in CL and implemented this approach three to four times a week for 15 weeks. The results showed a significant but small effect of CL on children’s social acceptance, but no significant effect on children’s friendships and perceptions of classroom relationships. The degree of CL implementation had effect on children’s social acceptance, but the effect was not consistent across social acceptance measures as a friend or a groupmate. Thus, it can be concluded that CL, conducted with the length and intensity of this study, may not lead to substantial changes in the social inclusion of children with SEN. In future studies, more focus needs to be devoted to teacher implementation of the CL approach.

## Introduction

While educational policies around the world have become more inclusive (Ramberg and Watkins, 2020), the social inclusion of children with special educational needs (SEN) still constitutes an area of concern. This concern has been expressed in a number of studies showing that children with SEN are less accepted by their peers and have fewer friends than their non-SEN peers (Pijl et al., 2010; Nepi et al., 2015; Schwab, 2015; Avramidis et al., 2018; Banks et al., 2018). Moreover, children with SEN have fewer interactions with classmates (Koster et al., 2010) and spend less time with peers during school breaks than their non-SEN peers (Schwab, 2015). They express lower self-concept and self-perception of social integration than their non-SEN peers (Pijl et al., 2010; Schwab, 2015). It is alarming that these patterns of exclusion seem to persist over time, as children’s friendships tend to remain stable over time (Frederickson and Furnham, 2001; Frostad et al., 2011; Schwab, 2019). Thus, although children with SEN are educated alongside their peers, there seem to be few opportunities for social inclusion.

The explanations for the challenges of social inclusion may be related to individual and contextual factors. Based on an individual perspective, it is the lack of the necessary social interaction skills that may be the reason for exclusion. For example, aggressive behavior and difficulties with social skills have been identified as predictors of peer rejection (Cillessen and Mayeux, 2004; de Boer et al., 2013; Schwab et al., 2015). Based on a contextual perspective, it is the factors in students’ environments that may explain exclusion or inclusion. Such factors include peer attitudes (de Boer et al., 2013), classroom norms (Gasser et al., 2017, 2018), and lack of teacher support (Mikami et al., 2013). From this perspective, it is important to provide opportunities for meaningful peer contacts (Grütter et al., 2018; Pinto et al., 2019). Moreover, teachers may need to address peer attitudes and friendships by creating inclusive classroom norms, modeling positive peer relationships, and giving children positive feedback (Mikami et al., 2013; Huber et al., 2018). In light of the contextual perspective on inclusion, this study contributes to previous research by evaluating a cooperative learning (CL) intervention aiming to promote social inclusion in classrooms with SEN children.

### Cooperative Learning Approach

In the CL approach, children work in small heterogeneous groups structured to enhance the learning of all group members (Slavin, 2014; Gillies, 2016). Several CL methods vary with regard to their theoretical underpinnings and specific elements, such as reward or task structures (for a review, see Slavin, 1996). An approach that has gained popularity in recent years is Learning Together (Johnson et al., 1993, 2009). This approach aims to promote group cohesion by structuring group work according to five principles based on social interdependence theory. The first principle, positive interdependence, ensures that all group members are aware that they are dependent on each other’s efforts in completing a task–a single member of a group cannot achieve anything unless all its members do. The second principle, individual accountability, means that all the group members feel responsible for completing their share of the group work. Promotive interaction, the third principle, implies that children are given possibilities to interact to promote group work by giving each other help, support, and feedback. The fourth principle, social skills, entails explicitly teaching social skills and motivating children to use them in group work sessions. The final principle, group processing, involves reflecting on the group work to plan future group activities. The teacher’s task in the CL approach is to structure classroom activities regarding the five principles of CL, introduce the activities, and monitor and intervene in the group work when needed (Johnson and Johnson, 2008; Gillies, 2016).

Reviews of the CL approach consistently show that it is a promising instructional method. CL approach has yielded higher results on children’s achievement across a wide range of school subjects (Johnson and Johnson, 2002; Roseth et al., 2008; Kyndt et al., 2013). It has led to positive changes in children’s perceptions of peer support, interpersonal attraction and liking, and children’s prosocial behavior (Gillies and Ashman, 1997, 2000; Slavin and Cooper, 1999; Johnson and Johnson, 2002; Roseth et al., 2008). However, the CL approach for the social inclusion of children with SEN is less researched. In a recent review of the effects of CL on the social inclusion of children with SEN, Garrote et al. (2017) identified six studies that included both CL and group activities in general, such as peer tutoring or support groups. According to the authors, the number of methodologically sound studies is still too small to conclude the effectiveness of group activities in promoting the inclusion of children with SEN.

### CL Approach and Social Inclusion of Children With SEN

The limited number of studies that have evaluated the effect of the CL approach on social inclusion of children with SEN show that this approach leads to increases in social acceptance and prosocial group behaviors (Putnam et al., 1996; Gillies and Ashman, 1997, 2000; Jacques et al., 1998; André et al., 2011; Capodieci et al., 2019). The interventions in these studies varied in length, from 12 h to 7 months, and in intensity, from 90 to 180 min per week. Thus, there is evidence for the benefits of the CL approach for children with SEN, even in short duration interventions. However, none of the studies evaluated children’s perceptions of their classroom relationships due to instruction according to the CL approach. In this study, the effects of the CL approach are evaluated on peer acceptance, friendships, and children’s own perceptions of their classroom relationships.

While there is evidence on the effect of the CL approach on inclusion, less is known of the conditions under which this approach has the intended effect. The social interdependence theory, a premise of the CL approach, posits that structuring tasks for positive interdependence among group members gives rise to psychological processes of caring for one’s group members and readiness to invest energy into others than oneself (Johnson and Johnson, 2009). However, this assumption was challenged by Slavin (2014), who pointed out that it may be a necessary but not sufficient condition for cooperation. Slavin (2014) proposed that group members may need additional motivational incentives to cooperate effectively, and that change as a result of such an intervention may take time.

Research on social inclusion may also add important explanations to the complexity of the relationship between the CL approach and social inclusion. In children’s decisions of whom to include, they appear to be balancing concerns of group functioning and moral issues of fairness and justice (Gasser et al., 2014). Peer group norms may influence these decisions. For example, under competitive classroom norms, group functioning concerns may weigh more, and peers may justify excluding a child with learning or behavior difficulties based on concerns of their group’s functioning (Gasser et al., 2017). The question is whether these processes take place in studies of the CL approach in heterogeneous groups, in which differences in academic status among members have led to problems with the participation of low-performing children (Cohen, 1994; Mulryan, 1995). If so, teachers may need to pay specific attention to creating inclusive classroom norms when using the CL approach.

Specific arrangements to promote group cooperation have been described in the CL approach studies and social inclusion. Different parts of a task were assigned to individual group members, or complementary roles were introduced (Jacques et al., 1998; André et al., 2011). For example, in a study by André et al. (2011) on CL’s use in physical education, goal interdependence was ensured by aggregating team results by adding up each team member’s score.

In addition to creating tasks conducive to cooperation, separate training in social and group work skills has proved to be important (Gillies and Ashman, 1997, 2000; Baines et al., 2015; Capodieci et al., 2019). In a series of studies, Gillies and Ashman (1997, 2000) had compared gains in children’s cooperative behaviors in two groups–children who participated in group work and children who, before the group work, participated in two training sessions with a focus on group work skills. As a result, the children in cooperative groups with additional training in group work skills showed more cooperative behavior and were more responsive to children who needed help. However, these benefits were not evident for children with learning difficulties, who improved only in on-task behavior. To conclude, merely assigning students to groups may not lead to social inclusion. Instead, it requires effort from the teacher to promote social skills and positive interdependence among group members.

### Implementing the CL Approach–Teachers’ Role

Teachers may find it challenging to implement the CL approach in their everyday instruction. In a survey by Abrami et al. (2004), 61% of teachers reported that they used CL, but a closer look into the teacher-reported practices revealed a gap between the recommendations and the actual classroom practices. Only half of the teachers in the study reported structuring group activities with principles of CL in mind. In a study by Lopata et al. (2003), professional development in CL was only associated with an increase in practices to support individual accountability, but not positive interdependence. The authors pointed to a need for greater attention to the elements that promote cooperation, such as positive interdependence, promotive interaction, and group processing. Difficulties in teacher implementation of CL have been confirmed in recent studies. Gillies and Boyle (2010) found that, although teachers had positive experiences of CL, they also experienced difficulties in responding to children socializing and not working, in managing the time effectively, and setting aside time for preparation. Buchs et al. (2017) reported that teachers perceived implementing CL as difficult, especially with regard to assessing children’s work and embedding CL within the curriculum. CL, as a practice, requires a profound change in teacher practices from teacher-led to child-centered pedagogy (Hennessey and Dionigi, 2013; Ghaith, 2018). Some researchers propose that to promote student cooperation, teachers need to cooperate with their colleagues, thus arguing for whole-school approaches in the implementation of CL (Sharan, 2010; Jolliffe, 2015).

The teacher’s role in the CL approach is central but intricate. Training in CL renders changes in the teacher’s role from modeling and providing direct instruction to one of monitoring and scaffolding group work (Blatchford et al., 2006; Gillies, 2016). It is generally acknowledged that less teacher involvement in group work is associated with greater autonomy (Lin et al., 2015). However, the teacher plays a profound role in framing the expectations of standard rules and norms in the classroom group work (Baker et al., 2017). These expectations may be communicated by asking and responding to questions or giving explanations (Webb et al., 2006). Furthermore, teacher guidance for group work may include prompting, modeling, and praising successful group processes (Lin et al., 2015). Thus, the teacher’s role includes the *a priori* structuring of group work for cooperation; following up on the social rules for interaction, and intervening when groups encounter problems. Regarding the reported challenges in implementing the CL approach, the teachers’ role in implementing the CL deserves careful attention in discerning the effects of this approach for the social inclusion of children with SEN.

### The Present Study

Concerning the need to study interventions that may promote social inclusion for children with SEN (Garrote et al., 2017), this study aims to contribute to previous research by investigating the effect of CL on social inclusion in classrooms in which children with SEN are educated alongside with their peers. Following a view of social inclusion as a multidimensional construct (Koster et al., 2009), the study focuses on three aspects of inclusion, peer acceptance, friendships, and children’s perceptions of classroom relationships. Also, concerning challenges in implementing the CL approach (Gillies and Boyle, 2010; Buchs et al., 2017), a special focus is devoted to the teacher implementation of the CL approach. The research questions are:

(a) What is the effect of CL approach and the effect of degree of CL implementation on children’s perceptions of classroom relationships?
(b) What is the effect of CL approach and the effect of degree of CL implementation on children’s social acceptance and friendships?

## Materials and Methods

A cluster-randomized experimental design was used to investigate the effects of CL on children’s social inclusion. The study was conducted per the guidelines for good research practices stipulated in the European Code of Conduct for Research Integrity (All European Academies [ALLEA], 2017). Before starting the study, ethical approval (Dnr 2017/372) was obtained from the Swedish National Ethical Committee.

### Participants

The study’s participants were 56 teachers of 958 fifth graders, aged 11 (498 boys and 460 girls). After recruitment and the submittal of informed consent by the teachers, the children, and their parents, the teachers and their classes were randomly assigned to intervention and control groups. Furthermore, to ensure equal prerequisites in the intervention and control groups, before the randomization, the teachers were divided into three groups based on their reports of previous knowledge and experience of CL. These groups were: (a) having knowledge and experience of CL, (b) having some knowledge or experience of CL, and (c) having no knowledge or experience of CL. The randomization was conducted within each group. Teachers working at the same school were randomized to the same group. As seen in Table 1, an approximately equal proportion of teachers in the intervention and control groups had knowledge and experience of CL.

**TABLE 1:** Background characteristics of classes and teachers in intervention and control groups.

	CL group	Control group
Number of teachers at recruitment	27	28
Number of children at recruitment	463	495
**Teacher knowledge and experience of cooperative learning**		
Knowledge and experience	9	6
Some knowledge	9	8
No previous knowledge	9	9
Mean number of students per class	27	27
**Proportion of children with SEN per class**		
Min	0.04	0.04
1st quartile	0.17	0.27
2nd quartile	0.27	0.33
3rd quartile	0.33	0.36
Max	0.60	0.58

Further, as seen in Table 1, the average class size was 27 children, and in 75% of classes, the proportion of children with SEN was 33–36%. In Sweden, the identification of special needs is not dependent on a medical diagnosis. Instead, it is up to the teachers and school welfare teams to determine which children are entitled to special support (Swedish National Educational Agency [SNAE], 2014). Children can receive two types of special support: extra adaptations and special support, documented in an individual educational plan (IEP). Thus, the proportion of children with SEN include those in need of extra adaptations and those who received support. Owing to regulations on the protection of individuals (SFS 2009:400, 2009), the information on children’s need for special support or type of special needs on an individual or school level is not accessible to researchers. Therefore, a special letter of consent was sent to children’s parents, asking them to approve teachers’ providing the researchers with information on their child’s need for special support. As the parents of only 12 students gave their approval for this, information on special support was retrieved through teacher reports on the class level.

Power analyses showed a need to recruit 51 classes and 1,020 children, based on an expected effect size of 0.30 and power of 80%, with an expected amount of 20 children per class and an intraclass correlation of 0.10. Therefore, 56 teachers, responsible for 1,169 fifth graders in 52 classes at 28 schools, were recruited. However, informed consent was obtained from the parents of only 958 children (463 in the intervention group and 495 in the control group). Furthermore, the attrition rate for pre- and post-measurement was 35% for perceptions of classroom relationships, so that 624 children were included in the analyses upon listwise deletion. For measures of peer acceptance and friendships, the attrition was 28%, resulting in analyses of data from 689 children. The reasons for attrition were teacher dropout from the study due to sick leave and personal circumstances (five teachers in the intervention group and two teachers in the control group). In addition, some teachers did not send the questionnaires to the researchers as intended, and some children were absent on the day of data collection.

In Appendix Tables A1, A2, the groups of children with missing values at pre- and post-measurement are compared with the children who participated in the study at both pre- and post-measurement. Comparisons between the groups were made using independent samples *T*-tests. Analyses revealed some degree of attrition bias. For children’s perceptions of classroom relationships, the children with missing values at pre- or post-measurement rated academic and personal support from their classmates and cohesion in a class lower than did the children who participated in both pre- and post-measurement. Regarding social acceptance, children in the control group who dropped out of the study at post-measurement, received more most liked nominations than the children who did not drop out. For friendships, significant differences between the groups were found in the intervention group. Children with missing values at pre- and post-measurement had significantly fewer reciprocated nominations than the children in the study.

### Intervention

Teachers in the intervention group received 5 days of training in the CL approach, divided into three phases. In the first phase, a 2-day training focused on the five principles of the CL approach, the creation of heterogeneous groups, and group-building activities. The participating teachers created lesson plans for activities that embedded the five principles of CL. The first phase of training lasted for 7 weeks. This phase also included a classroom visit for each teacher, during which researchers observed one lesson and provided feedback. During the school visit, the data on the quality of implementation of CL were collected.

The second phase of the training focused on embedding the CL principles in mathematics and literacy curriculum activities during 2 days of training. The researchers elaborated scripts of activities in mathematical problem-solving and reading comprehension, incorporating the five principles of CL. The second phase lasted for 6 weeks and included a classroom visit and personal feedback to each teacher, which also served as a data collection point. The third phase encompassed 1 day of training, scheduled 2 weeks before the end of the study. The training was based on the classrooms’ observations and focused on the CL approach’s theoretical foundations and additional activities to promote student interaction in groups.

In all the phases, in accordance with previous research on the implementation of CL (Jolliffe, 2015), the teachers were invited to participate in the activities they were to conduct with their children. The teachers were supported with training materials describing the theory behind CL, and activities and strategies aligned with its five principles, positive interdependence, individual accountability, promotive interaction, teaching social skills, and group processing (Klang et al., 2018). Based on the Learning Together approach (Johnson et al., 1993, 2009), the training materials were developed in cooperation with four teachers and 90 children who were not participants in the study.

The five principles of cooperation were ensured in all the activities. To promote positive interdependence, the teachers devoted time to group-building activities. After the teachers formed the groups, the children created a name and logo for them. The teachers introduced each lesson with their group’s common goal and emphasized group performance rather than individual performance. Tasks were structured to ensure that the children would be dependent on each other’s information or knowledge. For individual accountability, the tasks were introduced by emphasizing that each group member is responsible for the group’s work. At the end of each lesson, the children were asked to report on a task’s results. They did this either individually or rotated between different groups where a group member reported the results of their group’s work to the other groups. For promotive interaction, the teachers ensured that the children were seated near each other and shared the task’s materials.

To promote social skills, the teachers introduced one or two social skills per lesson. The same social skill was focused on for 1 or 2 weeks. The social skill was visible on the whiteboard, and activities to practice it were conducted. The teachers encouraged the children to practice their social skills and praised them when they did. For group processing, the teachers allowed time to evaluate the groups’ collaboration and decide on future strategies at the end of each lesson. The teachers worked on CL 3 to 4 days a week for 15 weeks. The groups were heterogeneous in terms of academic and social abilities, and the group composition alternated every 4 weeks.

#### Implementation of the CL Approach

Data on the fidelity of implementation included both adherence and dosage, in accordance with standards for evidence-based practices (Council for Exceptional Children, 2014). Data on implementation related to dosage was collected through teacher self-reports. Five times during the intervention period, the teachers were asked to provide information on the amount of time they devoted to CL over 2 weeks. Data on the quantity of implementation showed that in 21 of the 27 classes, the teachers reported having applied CL at least three to four times a week and in two classes in less than three to four lessons; no information was provided for the remaining four classes. Data on adherence were collected through observations using an observation protocol based on the principles of CL (Johnson et al., 2009). The observations revealed that most teachers implemented the CL intervention consistently through the intervention period, although the degree of implementation varied (see “Measures” section).

#### Control Condition

Teachers in the control condition were instructed to teach as usual. Due to a lack of time and resources, the observations in the control condition could not be conducted. To ensure that the teachers were interested in participating in the project, they received two lectures on mathematical problem-solving and reading comprehension despite being part of the control group. No cooperative activities were held.

### Measures

According to a broad definition of social inclusion (Koster et al., 2009), data on children’s social acceptance and friendships were collected through peer nominations. The children’s perception of classroom relationships was measured using the Classroom Life Instrument (Johnson and Johnson, 1983). Data on teacher implementation of the CL approach were gathered through lesson observations during school visits.

#### Perceived Classroom Relationships

The Classroom Life Instrument (Johnson and Johnson, 1983) consists of 16 separate scales aiming to explore children’s perceptions of classroom relationships. The instrument has shown acceptable reliability and validity (Johnson et al., 1983; Bertucci et al., 2016). Four scales from the instrument were used in this study. Two scales, Peer Personal Support (four items) and Peer Academic Support (five items), were used to measure children’s perceptions of peer support. Two indicators of overall classroom climate, related to inclusion, were added: Class cohesion (five items) and Valuing heterogeneity (four items). A five-step Likert scale was used, ranging from 1 (“Completely false”) to 5 (“Completely true”). The Student Academic Support scale includes four items focusing on children’s perceptions of peer support and how much peers care about their classmates’ learning, schoolwork, and school attendance. The Cronbach’s alpha value for this scale was 0.748. The Student Personal Support scale includes five items encompassing children’s perceptions of appreciation, friendship, and care from peers. The Cronbach’s alpha value for this scale was 0.841. The Class Cohesion scale includes five items on whether all children in the class are friends and know each other well, and a reversed question on feelings of loneliness. The Cronbach’s alpha value for this scale was 0.678. The Valuing Heterogeneity scale includes four questions on whether children appreciate learning from children who are different and if they perceive it as fun to work with and learn from them. The Cronbach’s alpha value for this scale was 0.739. The items in the four scales were translated and back-translated by two researchers following guidelines for cultural translation and adaptation (Brislin, 1970). The scales were pilot-tested with four teachers and 90 children in fifth grade prior to their use in the study. The pilot study showed that all the items in the scales were easy for the children to understand and respond to.

#### Peer Acceptance and Friendships

Peer social acceptance was investigated through most liked peer nominations, and peer friendships were calculated through reciprocal nominations. Negative nominations were avoided due to ethical considerations as well as the teachers’ and parents’ concerns regarding the effect of negative nominations on the children’s relationships (Child and Nind, 2013). Furthermore, the nominations were not limited to a certain number of children in a class, as unlimited nominations have higher ecological validity (Avramidis et al., 2017; Cillessen and Marks, 2017). Therefore, all children in a class could be selected, in accordance with the method used by Roistacher (1974). A matrix with two columns (“Who would you like to be friends with?” and “Who would you like to work in a group with?”) and rows representing the names of all the children in the class was used. The data for the students without parental consent were not included in the analyses. However, nominations from all submitted questionnaires in the classroom were included, when counting the nominations’ proportion for the students with parental consent. Proportion scores, calculated by dividing the number of nominations by the number of nominators, were used in the analyses (Velásquez et al., 2013).

#### Degree of CL Implementation

During the first school visit, the main author and a research assistant conducted observations in 14 classes. Inter-rater reliability, counted by dividing the number of agreements by the total number of ratings, was 76%. The observation protocol included eight domains, including the introduction of knowledge and social goals, ensuring the five principles of cooperation, and seating arrangements (see Table 2). As seen in the table, the observations were rated using a three-step scale: 0 (“Not present”), 1 (“Minimal requirements for implementation fulfilled–partial implementation”), and 2 (“All the requirements for implementation are fulfilled–full implementation”). The definitions of the three steps are presented in Table 2.

**TABLE 2:** Data on implementation adherence.

	School visit 1 (*n* = 21)			School visit 2 (*n* = 20)		
	0	1	2	0	1	2
Knowledge goal 0 not present; 1 the goal is presented by the teacher and is visible on the whiteboard; 2 the knowledge goal is formulated as a common goal, e.g., “all group members should…,” and is followed up.	0	8	13	2	9	9
Social goal 0 not present; 1 social goal is presented by the teacher and is visible on the whiteboard; 2 social goal is followed up during the lesson.	4	9	8	5	10	5
Social skills 0 not present; 1 social skills are presented by the teacher or are visible on the whiteboard; 2 social skills are given profound attention, and are modeled and reinforced during the lesson.	4	8	9	4	9	7
Promotive interaction 0 not present; 1 students are seated near each other and share materials; 2 students are seated near each other and the task requires cooperation.	3	9	9	1	8	11
Positive interdependence 0 not present; 1 group work is structured to strengthen cooperation, groups have identities, and group members have complementary roles; 2 the teacher follows up on the group’s common goal and encourages cooperation during the lesson.	1	16	4	3	11	6
Individual accountability 0 not present; 1 teacher introduces the task by saying that individual students will be asked to report; 2 teacher both introduces and follows up on the task either by asking individual students or by asking students to present the results to other groups.	7	11	3	4	6	10
Group processing 0 not present; 1 time is given for a short evaluation of the group work, e.g., voting; 2 time is given for a longer evaluation and reflection on the group work.	9	12	0	10	8	2
Seating arrangements 0 not present; 1 students are seated for the task; 2 permanent seating arrangements in groups.	0	9	12	0	7	13

A rating of 1 was assigned when the dimension was present at the beginning of the lesson, while a rating of 2 was assigned when the dimension was focused from beginning to the end of the lesson. For example, concerning the dimension of social skills, a rating of 1 was assigned to lessons in which the teacher introduced a social skill at the beginning of the lesson. A rating of 2 was assigned to lessons in which the teacher introduced the skills, explicitly accentuated the social skills during the lesson or provided praise for using them. As seen in the table, most of the teachers implemented six of the eight dimensions at least at a minimal level throughout the intervention period. It is also important to note that the lessons varied in the implementation of the dimensions, and there were no lessons in which all dimensions were not fully implemented.

As seen in Table 2, two dimensions–individual accountability and group processing–were not fully implemented by the teachers. For individual accountability, a higher number of teachers implemented this dimension during the second school visit. For group processing, the number of teachers who implemented this dimension was relatively low during the whole intervention period. In addition, as seen in the table, fewer teachers fully implemented all the dimensions, indicating that they introduced the dimensions but did not follow up and use them throughout the lesson. The data on implementation were aggregated to investigate the effect of the degree of teacher implementation of CL on children’s outcomes. The classes in which the aggregated ratings for the eight dimensions were lower than 16 for two observations were assigned the category “partially implemented,” while the classes in which the aggregated ratings exceeded 16 were assigned category “fully implemented.” These categories were further used in quantitative analyses of the effect of CL on children’s ratings of classroom relationships, social acceptance, and friendships.

### Statistical Analyses

Multiple multilevel regression analyses with a two-level structure were conducted (Twisk, 2006). Multilevel regression analyses allowed to account for the nested structure of the data, in which students’ responses were nested within their classrooms. The analyses were performed in R software, lme4 package, and children’s classrooms were considered a level 2 variable. The assumptions of normality of residuals, controlled by visual inspection of quantile-quantile plots, were met for all the variables. The number of outliers, which had a studentized residual value greater than ±3 varied from 0 to 15. In a case-by-case inspection, outliers with a value of Cook’s distance larger than 1 were deleted. Regression analyses were run on data, including and excluding outliers. As the results of the analyses did not differ, the decision was made to keep the outliers. The assumption of multicollinearity was met as variance inflation factors (VIF) were not greater than 10. The missing data were handled by listwise deletion, in which all the cases with missing values at pre- or post-measurement were deleted prior to the analyses.

Two multilevel models were used in the analyses. In the first model, students’ ratings of perceived classroom relationships as well as scores on social acceptance and friendships were studied as a function of time (pre- and post-measurement) and group belonging (intervention and control group), including an interaction between time and group. To account for variation in children’s pre-test scores within classrooms and individuals, two random intercepts were included, varying by class and individuals’ identification codes. When a random slope was added to the model, the random effects parameters could not be estimated, owing to a limited number of cases. Therefore, a decision was made to report the results of a model with two random intercepts, accounting for variation in students’ pre-test scores across classrooms. The second model was similar to the first model, except that the group belonging variable had three levels (control, CL partially implemented, and CL fully implemented). This was done to investigate the effect of the degree of implementation on children’s outcomes.

## Results

### What Is the Effect of the CL Approach and the CL Degree of Implementation on Children’s Perceptions of Classroom Relationships?

The results of multiple multilevel regression for children’s ratings of perceived classroom relationships are presented in Table 3. As seen in Table 3, in the first regression model, including the CL group and control group, the regression analyses’ results are not significant. Thus, it is not possible to conclude that being part of an intervention group using CL could be associated with higher ratings in children’s classroom relationship perceptions. In addition, Table 3 reports the results of the second regression model, including the control group and CL approach, partially and fully implemented. As seen in the table, the results are not significant, which indicates that the degree of implementation did not affect children’s perception of classroom relationships. Overall, the results show that the CL approach did not affect children’s perceptions of classroom relationships, regardless of whether the CL approach was fully implemented.

**TABLE 3:** Mean scores (standard deviation in parentheses), unstandardized multilevel regression estimates, and intraclass correlation coefficients for children’s ratings of perceived classroom relationships.

	CL (259)		Control (365)		Multilevel regression estimates			ICC
	Pre	Post	Pre	Post	Model 1	Model 2		
					CL: control b (95% CI)	Partial CL: control b (95% CI)	Full CL: control b (95% CI)	
Peer Academic Support	3.45 (0.64)	3.57 (0.67)	3.58 (0.60)	3.67 (0.66)	−0.03 (−0.07 to 0.13)	0.01 (−0.12 to 0.15)	−0.04 (−0.08 to 0.16)	0.169
Peer Personal Support	3.80 (0.73)	3.88 (0.70)	3.93 (0.68)	3.97 (0.69)	−0.04 (−0.06 to 0.14)	−0.05 (−0.18 to 0.08)	0.12 (−0.01 to 0.24)	0.089
Class cohesion	3.85 (0.67)	3.89 (0.64)	3.99 (0.67)	3.99 (0.68)	0.05 (−0.05 to 0.14)	0.02 (−0.09 to 0.15)	−0.07 (−0.05 to 18)	0.158
Valuing heterogeneity	2.95 (0.73)	2.95 (0.73)	2.94 (0.71)	2.90 (0.74)	0.04 (−0.08 to 0.17)	0.14 (−0.03 to 0.31)	−0.03 (−0.18 to 0.12)	0.019

*ICC, intraclass correlation coefficient; CL is used to denote cooperative learning intervention.*

### What Is the Effect of CL Approach and CL Degree of Implementation on Children’s Social Acceptance and Friendships?

The results of the two regression models for children’s social acceptance, studied by most liked nominations of their classmates as friends or groupmates, and children’s friendships, examined by reciprocated nominations, are presented in Table 4. As seen in the table, the CL approach had a significant effect on children’s social acceptance. However, the magnitude of regression coefficients is small, indicating that being part of a CL group could only lead to a small increase in most liked nominations. Furthermore, the results are significant in the second model, differentiating full and partial implementation of the CL approach from the control group. For most liked nominations as a friend, the effect is significant for the partially implemented CL approach compared to the control group. For most liked nominations as a groupmate, the effect is significant for a fully implemented CL approach. Thus, the second model results indicate that the degree of implementation might be important, but the results are not consistent across the two variables. For children’s friendships, measured through reciprocal nominations, there was no significant effect of the CL approach over the control group or significant effect of full or partial implementation of the CL approach compared with the control group.

**TABLE 4:** Mean scores (standard deviations in parentheses), unstandardized multilevel regression estimates and intraclass correlation coefficients for children’s social acceptance and friendships.

	CL (307)		Control (382)		Multilevel regression estimates			ICC
	Pre	Post	Pre	Post	Model 1	Model 2		
					CL: control b (95% CI)	Partial CL: control b (95% CI)	Full CL: control b (95% CI)	
**Social acceptance (most liked nominations)**								
As a friend	0.40 (0.18)	0.47 (0.19)	0.50 (0.16)	0.53 (0.17)	0.04** (0.02 to 0.06)	0.05** (0.03 to 0.08)	0.02 (−0.01 to 0.05)	0.478
As a groupmate	0.32 (0.18)	0.38 (0.19)	0.40 (0.20)	43 (0.19)	0.03** (0.01 to 0.05)	0.02 (−0.01 to 0.04)	0.04** (0.01 to 0.06)	0.280
**Friendships (reciprocated nominations)**								
As a friend	0.27 (0.17)	0.31 (0.20)	0.34 (0.18)	0.38 (0.20)	0.01 (0.01 to 0.03)	0.01 (−0.03 to 0.03)	0.02 (−0.01 to 0.05)	0.209
As a groupmate	0.16 (0.12)	0.19 (0.16)	0.22 (0.17)	0.24 (0.17)	−0.01 (−0.03 to 0.02)	−0.01 (−0.03 to 0.03)	−0.01 (−0.01 to 0.02)	0.274

**p ≤ 0.001. ICC, intraclass correlation coefficient; CL is used to denote cooperative learning intervention.

## Discussion

Having friends and positive relationships with peers is an important part of children’s school experiences, but research shows that it is not always the case for children with SEN (Pijl et al., 2010; Nepi et al., 2015; Schwab, 2015). Therefore, there is a need for research on interventions that promote social inclusion. This study, guided by research on the importance of contextual factors for inclusion (de Boer et al., 2013; Gasser et al., 2017), investigated the effects of the CL approach on social inclusion. While the CL approach is effective in whole-class approaches (Johnson and Johnson, 2002; Roseth et al., 2008), fewer studies have been conducted on its benefits for children with SEN (Garrote et al., 2017).

The study results showed that the CL approach had a small but significant effect on children’s social acceptance, but not on children’s friendships and perceptions of classroom relationships. Thus, the results corroborate previous findings on the effect of CL on social acceptance (Putnam et al., 1996; Jacques et al., 1998; André et al., 2011; Capodieci et al., 2019). Longitudinal studies reveal that patterns of friendships of children with SEN tend to remain stable over time (Frederickson and Furnham, 2001; Frostad et al., 2011; Schwab, 2019) and thus may be resistant to change. The CL intervention in this study lasted for 15 weeks. Hence, more time may be required to influence children’s friendships. Future studies are needed to extend CL interventions over longer periods of time.

The study results indicate that the CL approach, implemented with the duration and intensity of this specific study, does not lead to social inclusion. In previous research, unequal participation patterns were observed in heterogeneous groups (Cohen, 1994; Mulryan, 1995). These patterns may be exposed during group formation when roles and norms are established in cooperative groups. Children with SEN may be particularly vulnerable in these situations. Children’s inclusion decisions are complex, and they may be influenced by considerations of their group’s functioning and norms (Gasser et al., 2014, 2017). Thus, teachers may need to consider the classroom norms when using the CL approach and accentuate inclusive classroom norms, valuing diversity and equal participation.

The CL approach assumes that children develop positive experiences of group work through a feeling of interdependence, created through the five principles of the approach, including positive interdependence, individual accountability, promotive interaction, explicit instruction in social skills, and group processing (Johnson and Johnson, 2009). Studies of CL, focusing on social inclusion of children with SEN, reported on the need for additional training in social skills (Gillies and Ashman, 1997, 2000; Baines et al., 2015; Capodieci et al., 2019). Moreover, specific procedures to ensure positive interdependence were used by aggregating team results on individual scores (André et al., 2011). In this study, the implementation of the five CL principles was assured through activities and materials. Based on the lack of a significant effect on social inclusion, future studies of the CL approach may be necessary to further accentuate training in social skills and to promote positive interdependence among group members.

As highlighted above, implementing a CL approach appears to be a formidable task for teachers. Therefore, it is important to explore how the CL approach can be incorporated into the teachers’ everyday practices. In this study, the degree of CL implementation did not affect children’s perceptions of their classroom relationships or friendships. It had, though, a significant effect on children’s social acceptance as a groupmate. However, the observational data on implementation revealed that not all teachers fully implemented the CL approach in their classes. The dimensions of CL that were not sufficiently implemented were individual accountability and group processing. While individual accountability had been implemented by most teachers by the end of the intervention, not all the teachers in the intervention group devoted time to group processing. The groups need to evaluate their work and plan future actions to function well (Johnson et al., 1993, 2009). Given the importance of group processing, it is troubling that this element was not consistently used in the intervention. Teachers may struggle with the time needed to prepare lessons and fit the CL approach into the classroom curriculum (Gillies and Boyle, 2010; Buchs et al., 2017). Future studies should focus on how the CL approach can be fully implemented concerning teachers’ everyday practices.

Another aspect of the implementation of CL concerns the teacher’s role in CL. In this study, as seen from the observations of the teachers’ practices, they successfully introduced the principles of CL at the beginning of the lesson, but these principles were seldom followed up throughout the lesson. Previous research has emphasized the importance of teacher framing expectations for social rules and norms in group work (Webb et al., 2006; Baker et al., 2017). As the teacher’s role shifts from that of providing direct instruction to one of scaffolding group work, teacher guidance of group work through prompting, praising successful group processes, or modeling is essential (Blatchford et al., 2006; Lin et al., 2015). The identified challenges in CL implementation in this study suggest a need for further research on the teacher’s role in CL, including video observations of teaching and interviews with both teachers and children.

Previous studies have emphasized CL’s complexity, as it is not simply a technique but requires a shift from a teacher-led to a child-focused pedagogy (Hennessey and Dionigi, 2013; Ghaith, 2018). Thus, an intervention of 15 weeks may have been insufficient to give rise to these profound changes in teacher practices. Some researchers advocate whole-school approaches in the implementation of CL (Sharan, 2010; Jolliffe, 2015), arguing that to change the cooperation climate in the classroom, teachers need to change the way they cooperate with colleagues. In this study, individual teachers rather than schools were recruited for participation. Further studies on the implementation of CL may need to consider the importance of teacher teams in the implementation.

## Limitations

The present study has several limitations. Firstly, due to regulations concerning the protection of individuals in Sweden (SFS 2009:400, 2009), the data on individual children’s need for special support could not be disclosed without the children’s legal guardians’ permission. Upon the sending of an additional letter of consent, data on individual children’s need for special support was retrieved for only 12 children, thus leaving no space for meaningful investigation of the benefits of CL for these children. However, based on the teachers’ reports, 75% of the classes in the study had 33–36% children with SEN. So, although it is not possible to draw conclusions about the benefits of CL for individual children with SEN, the study contributes to research on the use of CL in classes with SEN children.

The second limitation concerns the teachers in the study. Upon recruitment, it was clear that the teachers were interested in using the CL approach for inclusion. Teachers in both the intervention and the control group had some knowledge and experience of CL. Although the teachers in the control group were not encouraged to use CL, their teaching may have contained elements of it. Due to a lack of time and resources, data on teaching in the control group were not collected, thus constituting a threat to the study’s internal validity.

The third limitation concerns attrition bias in the study, which may also have influenced its internal validity. Analyses revealed significant differences between the groups of children with missing values and the groups of children who participated from the beginning to the end of the study: those who dropped out of the study rated their classroom relationships lower and had fewer friendships. Attrition bias may indicate that introducing CL in classes characterized by lower cohesion, less positively perceived classroom relationships, and fewer friendships at the start may be more difficult and lead to participant dropout.

Finally, the conclusions from the study may be limited due to the choice of outcome measures. In this study, only three of four dimensions of social inclusion (Koster et al., 2009) were investigated: peer social acceptance, friendships, and perceptions of children’s classroom relationships. Data on peer interaction dimension were not collected. Observations of interactions among the children in the classrooms and during breaks might have rendered more accurate and ecologically valid measures.

## Implications

Despite reforms to ensure access to mainstream schools for children with SEN, social inclusion remains a challenge. This study focused on CL as an intervention to improve social inclusion in classrooms with students with SEN. It was assumed that this method could alternate the patterns of peer relationships in the classrooms by engaging children in heterogeneous groups in which the work was structured following the principles of productive collaboration. Although the study results showed small to non-significant effects of the CL approach on social inclusion, they may–with reservations regarding the study’s limitations–offer important insight into when an intervention to support inclusion is not sufficient. The results indicate that, merely using CL approach may not lead to profound changes in social inclusion. In order for CL to be an effective practice, there is a need to look into teachers’ everyday practices of CL in classrooms to understand how and why CL may promote social inclusion. In this regard, it is especially important to study how teachers can create optimal conditions for cooperation in heterogeneous groups.

## Data Availability Statement

The raw data supporting the conclusions of this article will be made available by the authors.

## Ethics Statement

The project was approved by Uppsala Ethical Regional Committee, Dnr. 2017/372. Written informed consent to participate in this study was provided by the participants’ legal guardian/next of kin.

## Author Contributions

NK and GL has participated in data collection, data analysis, and data interpretation. IO and JW has participated in data analysis and data interpretation. NF has participated in data collection, in the design of the intervention study, and in data interpretation. CN has participated in the design of the project and in data interpretation. All authors contributed to the article and approved the submitted version.

## Conflict of Interest

The authors declare that the research was conducted in the absence of any commercial or financial relationships that could be construed as a potential conflict of interest.

## Appendix

**TABLE A1:** Students ratings of perceived classroom relationships in questionnaire Classroom Life Instrument, reported separately for those with missing values at pre-measurement, post-measurement and remaining participants.

	Missing at pre-measurement		Missing at post-measurement		Remaining participants	
	CL (*n* = 95)	Control (*n* = 54)	CL (*n* = 109)	Control (*n* = 76)	CL (*n* = 259)	Control (*n* = 365)
Academic support T1			3.29* (0.70)	3.54 (0.57)	3.45* (0.64)	3.58 (0.60)
Personal support T1			3.57* (0.86)	3.75 (0.75)	3.80* (0.73)	3.93 (0.68)
Class cohesion T1			3.63* (0.80)	3.82* (0.64)	3.85* (0.67)	3.99* (0.67)
Valuing heterogeneity T1			2.88 (0.77)	2.84 (0.68)	2.95 (0.73)	2.94 (0.74)
Academic support T2	3.60 (0.70)	3.40** (0.73)			3.57* (0.67)	3.67 (0.66)
Personal support T2	3.90 (0.79)	3.80 (0.85)			3.88 (0.70)	3.97 (0.69)
Class cohesion T2	3.85 (0.69)	3.91 (0.78)			3.89 (0.64)	3.99 (0.68)
Valuing heterogeneity T2	3.11 (0.73)	3.06 (0.79)			2.95 (0.73)	2.90 (0.74)

*Values are mean values (standard deviations) unless stated otherwise. Significant differences between the remaining participants and the groups with missing values, as studied by Independent samples T-tests at significance levels *p = 0.05, **p = 0.01.*

**TABLE A2:** Students’ peer nominations, reported separately for those with missing values at pre-measurement, post-measurement, and remaining participants.

	Missing at pre-measurement		Missing at post-measurement		Remaining participants	
Peer nominations	CL (*n* = 54)	Control (*n* = 29)	CL (*n* = 102)	Control (*n* = 84)	CL (*n* = 307)	Control (*n* = 382)
Peer nominations as friend T1			0.42 (0.16)	0.53* (0.14)	0.40 (0.18)	0.50* (0.16)
Peer nominations as group-mate T1			0.31 (0.16)	0.35 (0.17)	0.32 (0.18)	0.40 (0.20)
Reciprocated peer nominations as a friend T1			0.27*** (0.16)	0.32 (0.23)	0.35*** (0.18)	0.25 (0.17)
Reciprocated peer nominations as a group-mate T1			0.16*** (0.13)	0.15 (0.12)	0.23*** (0.17)	0.14 (0.12)
Peer nominations as friend T2	0.44 (0.17)	0.52 (0.13)			0.47 (0.19)	0.53 (0.17)
Peer nominations as group-mate T2	0.33 (0.16)	0.44 (0.17)			0.38 (0.19)	43 (0.19)
Reciprocated peer nominations as a friend T2	0.26*** (0.17)	0.31 (0.22)			0.41*** (0.19)	0.30 (0.19)
Reciprocated peer nominations as a group-mate T2	0.10*** (0.08)	0.19 (0.22)			0.30*** (0.16)	0.18 (0.16)

*Values are mean values (standard deviations) unless stated otherwise. Significant differences between the remaining participants and the groups with missing values, as studied by independent samples T-tests at significance levels *p ≤ 0.05, ***p ≤ 0.001.*
